# A Digital App to Aid Detection, Monitoring, and Management of Dyslexia in Young Children (DIMMAND): Protocol for a Digital Health and Education Solution

**DOI:** 10.2196/resprot.9583

**Published:** 2018-05-17

**Authors:** Mariam R Sood, Annet Toornstra, Martin I Sereno, Mark Boland, Daniele Filaretti, Anuj Sood

**Affiliations:** ^1^ Synergation Limited Middlesex United Kingdom; ^2^ Work and Social Psychology Faculty of Psychology and Neuroscience Maastricht University Maastricht Netherlands; ^3^ Department of Psychology College of Sciences San Diego State University San Diego, CA United States

**Keywords:** dyslexia, digital health, early education, special education needs, study skills, EdTech, serious games

## Abstract

**Background:**

Dyslexia, a specific learning difficulty and a disability as defined in the Equality Act 2010, is a lifelong condition that affects a child from the start of education. Dyslexia is characterized by difficulties in language processing (reading, spelling, and writing) which do not correspond with the child’s general intellectual abilities. Although dyslexia cannot be cured, there is a consensus that interventions are more effective and have greater impact the earlier they are administered. Effective interventions start with diagnosis. Currently, formal diagnosis requires an assessment by a dyslexia specialist or educational psychologist. These assessments are expensive and are not easy for a non-specialist teacher or parent to interpret. Consequently, formal assessments are normally performed at a much later age, when interventions are less likely to be effective. Combining the latest in scientific research, expertise of dyslexia practitioners and real-time interactivity facilitated by digital technologies, we aim to provide a cost-effective and convenient solution that focuses on early dyslexia detection and management.

**Objective:**

We discuss the rationale and protocol for the design and development of a digital health solution aimed at improving the early detection, monitoring and management of dyslexia (DIMMAND) in young children (4-8 years). The primary objective is to create a game-based digital solution aimed at children, parents, and teachers that firstly assesses, then monitors and manages progress in a convenient, cost-effective and private environment.

**Methods:**

The proposed solution will be designed and developed in phases. In the initial phase, the full functional specification of the games that constitute the app will be designed, together with the overall architecture of the solution. Prototype proof-of-concept implementation for few of these games, and commercialization strategies will also be developed. The follow-on phases will see the design implemented into a validated solution.

**Results:**

In the initial phase, we worked closely with dyslexia specialists, adult dyslexics, teachers of special-needs children, parents of dyslexic children, and senior dyslexia representatives for large organizations. These interactions provided insights into the range of language difficulties faced by dyslexics, which solutions are used by teachers and professionals, and an overall understanding of the market. We comprehensively defined the ethical, privacy, and data security issues. The detailed design spec of the games, the methodology to be followed to interpret the results, and flow diagrams illustrating how the game screens will be presented was completed. As proof of concept, a few reading, visual, and auditory games were developed and successfully tested by stakeholders on different digital devices. The stakeholders provided regular feedback and confirmed the viability of our game-based solution.

**Conclusions:**

DIMMAND has the potential to provide significant positive health care and economic impact. It is expected to reduce intervention costs, improve dyslexia detection at an early age and aid self-management.

**Registered Report Identifier:**

RR1-10.2196/9583

## Introduction

Developmental Dyslexia is a learning difficulty and a disability, as defined by the Equality Act 2010. It has serious short- and long-term effects that impact a child from the start of his or her education. Dyslexia is a lifelong condition [1-4], estimated to affect around 700 million people worldwide [2,5]. Dyslexia is characterized by difficulties in language processing (reading, spelling, and writing) which do not correspond with the child’s general intellectual abilities [3,6]. In addition, dyslexia occurs across a wide spectrum, which makes it difficult to identify suitable customized interventions [4].

The neural basis of dyslexia is contested. Though there are likely many neurobiological factors contributing to dyslexia, several prominent theories identifying the main causal role have been proposed. The earliest theory was that dyslexia results from putative visual deficits, giving rise to difficulties with the processing of letters and words [7-9]. In the 1980s, dyslexia was reconceptualized as a weakness in phonemic awareness or letter-sound mapping, the foundation of reading for alphabetic systems [10-15]. This is by far the most popular theory and forms the basis for the majority of commercial assessment tools. It is, however, challenged on the premise that a more basic auditory deficit-impairment in the perception of short or rapidly varying sounds is the root cause [16,17]. Recent research suggests that visual-spatial attention skills are an excellent predictor of preschool children’s future reading abilities [18,19]. Neuroimaging research in non-dyslexic adults shows widespread cortical activation during natural reading tasks, which overlap heavily with the visual maps that encode the visual space, auditory maps that encode sound frequencies, and the frontal regions of the brain [20,21]. Evidence from the scientific literature suggests that reading and writing difficulties can be caused by varied factors and successful interventions must therefore be customized to the individual’s specific difficulty [22].

As mentioned previously, it is likely that varied neurobiological reasons manifest as dyslexia. It is therefore crucial to identify the individual’s relevant strengths and weaknesses to provide the individual with effective customized support. Success with basic reading and writing is critical to overall success in life. Hence it is important to identify tailored strategies needed to help manage early learning processes that will ensure the individual receives the necessary attention applicable to their strengths and opportunities.

DIMMAND brings together all the scientific advances made in the field to date to provide a widely-available and cost-effective digital tool for the public use. We recognize the merit in each one of the above-mentioned theories, and acknowledge the need to bring them together under one coherent framework to detect the many different reasons why a child might be experiencing difficulty with reading and writing. The major goal of the project discussed in this paper is to devise a digital solution that will (1) help identify at a relatively young age (4-8 years) the nature of the literacy difficulty or difficulties the child experiences, and (2) propose tailored interventions to guide parents and less-specialized teachers to provide better support before the child experiences repeated failures.

The digital tool will feature innovative games that systematically explore a child’s abilities in different aspects of reading and writing and their corresponding abilities in purely visual and auditory perception. Another important objective of DIMMAND is to help teachers or parents identify the most effective way to help a child as quickly as possible. Firstly, this is achieved by involving them in the testing process and secondly by providing customized intervention options which can be put into practice immediately. A key aspect of this app will be the inclusion of automated interventions, derived from existing research and educational strategies, to help identify which options, if any, will be beneficial to the child. Where an intervention is found to be helpful to the child, it will be incorporated into the presentation of rest of the app.

In the UK, dyslexia assessments are not available free of charge from the National Health Service (NHS). A professional assessment of dyslexia is expensive, costing upwards of £350. This assessment generally involves a session with a psychologist or dyslexia specialist who uses a combination of standardized tests to check the child’s intelligence quotient (eg, Weschler intelligence scale for children [23]), reading, spelling, and writing abilities (eg, comprehensive test of phonological processing [24], wide-range achievement test [25], test of word reading efficiency [26]). Such assessments are carried out by educational psychologists or specialist teachers. Since it is not easy to diagnose with certainty a young child as dyslexic at the start of his or her education, these formal assessments are normally performed much later, after the child has been in school for several years and has presented with severe literacy difficulties. This is not ideal for a developing child, as it can affect self-confidence and may result in failure to fulfil their potential [6]. A few screening tools for young children are available such as Dyslexia Early Screening Test [27], Lucid Cops [28], and Dyslexia Quest [29].

DIMMAND differs from the existing solutions in two important ways. Firstly, the tests are structured in a bottom-up format and exploit the latest neuro-scientific evidence on dyslexia. Reading and writing are complex skills which involve visual, auditory, visuo-auditory, memory, and motor cognition. Our tests will systematically assess the various cognitive skills required for successful reading and writing. Secondly, inclusion of automated interventions enables the app to be more than a mere detection tool, by delivering dyslexia management at an individual level.

The rest of this paper presents the protocol for the proposed first phase of the project. In this phase, we will assess the feasibility of developing a digital solution for early identification of dyslexia. The key elements of the digital solution are: (1) to systematically assess the nature of literacy difficulties experienced by the child and (2) to propose tailored interventions.

## Methods

### Project Design

The proposed solution will be designed and developed in phases. In the first phase, the full functional specification of the test games that constitute the app were designed, together with the overall architecture of the solution. In addition, prototype proof-of-concept implementation for several of these games will be developed. The app design will be informed by insights provided by adult dyslexics, teachers and parents of dyslexic children, and by organizations and charities working in the field. The templates for user-engagement interviews are provided in Multimedia Appendix 1. Ethical and data management strategies have also been explored and will form part of the deliverables. Additional activities in this phase planned and underway include the development of a commercialization strategy for DIMMAND. In the follow-on phases, the design will be implemented into a validated solution.

### App Development

The app will be developed with Unity [30], one of the world-leading cross-platform game development frameworks. Well established best-practice for software engineering will be followed. For example, a Model-View-Controller layer [31] will be used on top of Unity’s built-in Entity-Component-System architecture [32] to maximize modularity and maintainability of the app.

This technology stack will allow our solution to be deployed and adapted to a variety of platforms including PC, Android, and iOS using a single code-base with minimal effort and without the need to write additional platform-specific code.

A user interface (UI) and story line will ensure the test presentation is engaging and stimulating for child users. The UI will be designed to be minimalistic, so as not to interfere with the testing itself, but at the same time it will be engaging to a young child.

**Figure 1:**
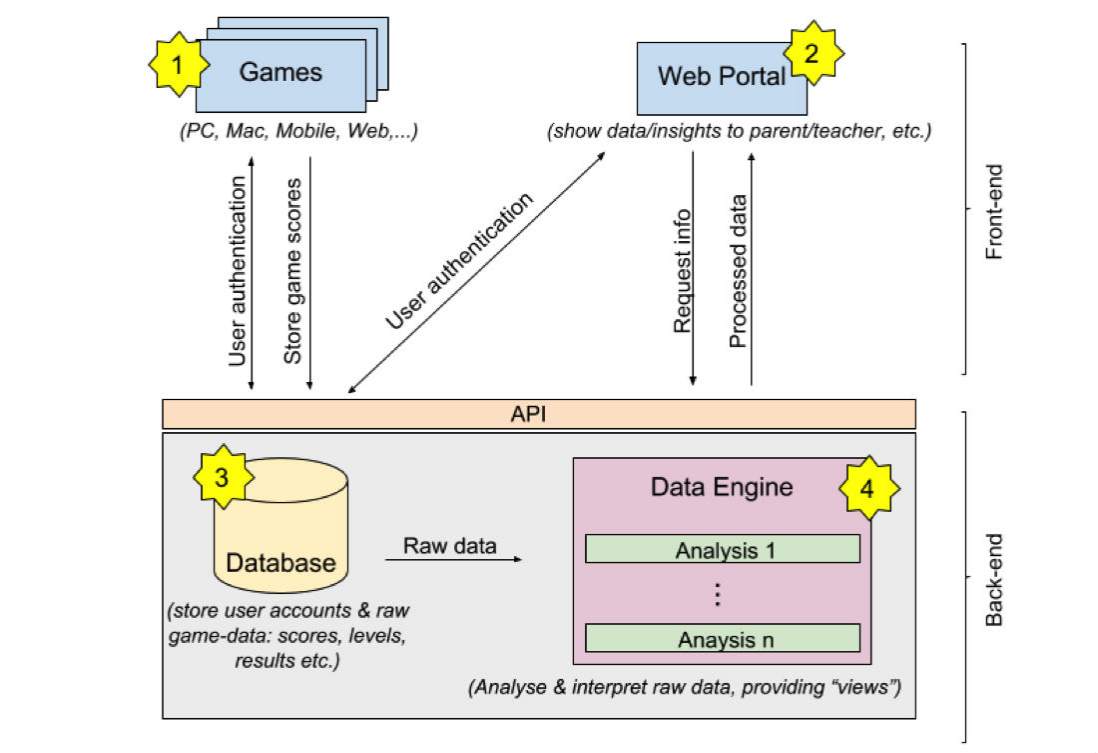
DIMMAND - System Architecture.

At a high level, the system will comprise of a front-end and a back-end (Figure 1). The front-end includes the user-facing portions of the system; namely the app itself (for use by children) and the web-portal (for use by teachers and parents). The back-end will store and process data and will be where other important data and intellectual property not included in the client software, such as test generation algorithms, is stored. Front-end and back-end are completely independent and will communicate via the Web.

### Expected Outcomes

During the first phase of the project, our Work Packages (WP) will deliver the following outcomes:

1. User Requirements Report (WP1): This will define the service and its underlying components. Stakeholders will be engaged in the design phase to understand the user’s experience of the technology.
2. Report on Ethics, Legal, Data Security and Privacy Considerations (WP1): This will explain how the following issues are managed: informed consent, consideration of legislation, secure processing of personal data, etc.
3. Reading, Visual and Auditory Games Specification document (WP2): A detailed spec of the games, which will be implemented. The games will systematically explore a child’s capabilities in various aspects of reading/writing as well as in visual and auditory perceptions
4. Mock-up of the app with few concept games (WP3): User interfaces will be designed to show possible game flows and pages. The concept games will be developed using the cross-platform Unity framework.
5. Business Plan (WP4): This will include details on opportunity, vision and aim, market analysis and horizon scanning, target customers, business model, financials and follow on funding/investment, strategic analysis, intellectual property protection, and team.

In the follow-on phases, the design developed during phase one will be implemented into a fully-fledged app and validated solution. This will include full-scale implementation of the games designed during phase one and include the design and implementation of the back-end database, data analysis, testing and validation with end-users, and productization of the solution.

### Problems Anticipated

The following risks have been identified for the successful deployment of the solution.

- Games development (High)_:_ There is a risk that our games will not engage young children sufficiently. Since the games are intended to test the skills a child needs to read successfully, there is a challenge in finding the right balance between functionality of the game and an engaging user interface. This is a key challenge and it will be addressed via iterative user feedback.
- Data residency, hacking and privacy (High)_:_ App security will be embedded within our architecture and then validated and tested. We will ensure that both the app and data are secure at rest, in transit or in use. We will comply with the Data Protection Act (and the General Data Protection Regulation [GDPR] from 25 May 2018).
- Technology is new to the environment (High)_:_ Understanding the unique strategies to help a dyslexic child is traditionally carried out by attentive teachers in one-to-one settings. Utilizing technology to assist with special-needs education is an innovative concept. Achieving widespread take-up and effective use by end-users will require change management to be adequately supported.
- Ethical issues of unintended consequences (Low)_:_ Mechanisms to audit and monitor for these issues—and to mitigate them if found—will be developed during the prototype-development stage.

### Ethics

For the initial phase of the project, user-engagement interviews were carried out to inform our approach to designing the test games. All interviewees who participated in the user-engagement phase of this project were given information about the project and their explicit consent was taken prior to the interview. The interview notes and transcripts were anonymized to safeguard the interviewees personal identity. The Caldicott principles [33] regarding patient-identifiable information will be continuously followed for the duration of the project.

Appropriate measures to address privacy and data security will be incorporated into our development plan. The architecture will embed app security and this will be validated and tested. This includes the design of our app and the selection of any servers (including cloud) with which the app will communicate. We will comply with the Data Protection Act from the moment data is obtained until the time when the data have been returned, deleted, or destroyed. Our servers will reside in the UK and European Economic Area (EEA) and no personal data will be transferred to a country of territory outside the EEA. Full compliance with the forthcoming GDPR and the existing Data Protection Act 1998 will be ensured. All ethical procedures in place will be detailed and documented as part of the deliverables for the first phase of the project, which will serve as the reference for the future development. Since the initial phase of the project did not involve research on human subjects, no ethical approval was necessary.

## Results

The first phase of the app development commenced in March 2017. The results of the first phase will be utilized for the full-scale development of the solution. Additional publication is expected in early 2019.

## Discussion

DIMMAND has the potential to provide a significant positive impact on health and education, and a positive economic impact. If each dyslexic child has access to a qualified, supportive, and attentive teacher, his or her potential to succeed in life is immense. However, unfortunately, that is often not the case. This makes the need for a widely available tool, such as the one proposed in this project even more urgent. This solution will assist a teacher or parent with no specialist knowledge of dyslexia to understand where the child is struggling (including whether it is a language-specific issue or related to general perception) and what kind of intervention will be most effective in helping the child. The games featured in the proposed app will be based on the range of scientific theories underpinning dyslexia. Overall, DIMMAND is expected to reduce intervention costs, improve dyslexia detection at an early age, and aid self-management.

## Appendix

Multimedia Appendix 1 User engagement interview questions.

## Abbreviations

EEA: European Economic Area
GDPR: General Data Protection Regulation
NHS: National Health Service
UI: user interface
UK: United Kingdom
WP: Work Package
