# Supplementary material for: A Digital App to Aid Detection, Monitoring, and Management of Dyslexia in Young Children (DIMMAND): Protocol for a Digital Health and Education Solution
Source: JMIR Res Protoc. 2018 May 17;7(5):e135. doi: 10.2196/resprot.9583 (PMC5981053; doi:10.2196/resprot.9583)
Supplement: Multimedia Appendix 1 [file resprot_v7i5e135_app1.pdf]

Appendix 1

Questions used for user engagement interviews

- **Adult dyslexics**
  1. How did you experience learning to read?
  2. Why did you feel that (experience of learning to read) was difficult?
  3. When did you first experience that?
  4. Did you feel you were different to your peers?
  5. When did you experience that feeling?
  6. What differences did you feel?
  7. How did you experience learning to write?
  8. How did you feel your spelling developed?
  9. What strategies helped, or what did you do to make it easier?
  10. What help did you get from the system?
  11. Did you feel that the system supported you?
  12. What would you recommend- how should a 5/6/7/9/11 year old child be helped?
  13. Are there still issues in reading, writing and spelling for which you found special solutions?
  14. How did you feel about learning other languages? How was that experience?
  15. Any differences between how you acquired other language(s) as compared to English?
  16. Anything else that hampered your progress?
  17. Any co-occurring difficulties?
  18. How was/is your auditory comprehension?
  19. Any other comments (on speech, writing etc.)

- **Teachers**
  1. To what extent do you feel prepared and educated to detect dyslexia?
  2. To what extent do you feel ready to help the students with dyslexia?
  3. What would you wish? In an ideal world, what would you offer the child?
  4. Do you use any software or other tools to screen the child, once a child is suspected of dyslexia?
  5. Once the child has been recognised by teachers, how long it takes within your school and your local region to get additional support?
  6. Do you feel there is a link with Math problems?
  7. Do you feel there is a link with motor or visuospatial problems, or any other problems?
  8. How do you perceive parents? Do parents have insight on the learning difficulties of the child?

- **Parents**
  1. When did you first notice something was different? And what did you notice?
  2. What did you observe in early learning process?
  3. What did you observe in learning to read?
  4. What did you observe in learning to write?
  5. How do you feel your child perceived going to school?

6. Did you observe any other issues with learning?
7. What strategies did you try with your child? And what worked?
8. What was the response from school?
9. Who took the initiative? School or yourself?
10. When did you start working with the school?
11. What testing has been done? And what reports have been helpful?
12. What support from the school has been helpful?
13. Does insurance pay for the testing or treatment?
14. Do they have access to help outside the school?
15. Retrospectively, How you would have helped your child in his/her early stages?

- **Organizations**
  1. What software do you use for your assessments?
  2. What do you think about the range of software (for dyslexia) that is available in the market?
  3. Your comments on automating interventions
  4. How well do you think you are helping dyslexics? How many teachers/schools are using your services?
  5. What do you think about the proposed solution?
  6. At around what age, children generally get diagnosed? If that age is late, what are the reasons?
  7. Your comments on the ethical issues surrounding dyslexia detection targeted at young children
  8. How well are teachers equipped for dealing with dyslexia?
  9. How well are parents equipped for dealing with dyslexia?
